# Intraosseous Vascular Access Device as a Transarticular K-wire Alternative in Mallet Finger Laceration

**DOI:** 10.5811/cpcem.2017.7.34811

**Published:** 2018-01-18

**Authors:** Scott B. Crawford

**Affiliations:** Texas Tech University Health Sciences Center El Paso, Department of Emergency Medicine, El Paso, Texas

## Abstract

Mallet finger is a common injury often treated without operative intervention. When there is concern for skin integrity or a large articular component is involved, simple operative repair may be needed. This has been performed with transarticular Kirschner wire (K-wire) placement. This case discusses the novel use of an intraosseous vascular access device (IOVAD) as a potential adjunct to stabilization and alternative to treatment with operative K-wire fixation. A 53-year-old man was successfully treated using the inner trocar of the EZ-IO® system for a mallet finger injury with laceration, shown in comparison with another standard manual pinning approach using an 18-gauge needle. An IOVAD can be used successfully as an alternative to K-wire placement in patients with mallet finger injuries.

## INTRODUCTION

Disruption of the extensor tendon at the distal interphalangeal joint (DIP) is common with lacerations due to the proximity of this structure to the skin surface. Two-thirds of extensor tendon lacerations are also associated with coincident damage to bone, skin or joint.^1^ The disruption of this tendon is traditionally referred to as “mallet finger.” Stress through forced flexion of an extended DIP causes disruption of the extensor mechanism often due to sports or work-related injury, for example a ball striking the extending finger of a basketball player.^2^ This injury pattern can cause deformity and disability if not treated appropriately. Most often it does not require surgical intervention. The severity of this injury and need for referral depends on the involved structures. There is debate as to the best technique, material and procedure to stabilize the injury to assure optimal healing, but initial splinting of the digit in extension for four to eight weeks is a clear consensus.^3^–^5^

Recommendations in the currently available literature regarding management of DIP extensor tendon injuries include use of basic aluminum and molded splint material, use of Kirschner wire (K-wire) and similar materials for stabilizing fracture fragments, bio-absorbable surgically implantable stabilization devices, screws and even arthrodesis.^6^,^7^ It has been recommended that use of pinning devices like the K-wire be implemented to allow for full extension of the joint to best protect the repair and allow monitoring of the skin.^1^,^8^ Care is needed when placing an appropriate splint due to concern for skin breakdown and tissue ischemia in dorsally applied splints.^4^ In addition to keeping tension off the disrupted tendon, open tendon repairs can be achieved with tenodermodesis (the suturing of tendon to the skin during healing).^3^

Orthopedists have access to all manner of tools and materials in the operating room. Emergency departments and urgent care centers, however, are limited to a much smaller inventory to cover a wider number of potential ailments. Appropriate care must therefore be rendered with what is available at the time of patient presentation, with the knowledge that definitive follow-up can be achieved within the next hours to days after initial stabilization.

One product gaining popularity in many centers that care for critically ill patients, especially pediatric populations, is the intraosseous vascular access device (IOVAD). These devices are designed for the cannulation of the medullary cavity of various bone sites during resuscitation for fluid and medication administration when standard intravenous access is not quickly available. This paper discusses a novel use for the inner stylet of an IOVAD needle to aid in the treatment of mallet finger. The device used in this case was the EZ-IO^®^ made by Vidacare^®^.

## CASE REPORT

A 53-year-old Hispanic man presented with dorsal laceration to his third and fourth DIPs with bleeding and loss of mobility. The patient was right-hand dominant, and just prior to arrival he sustained a crush/laceration injury at work to this hand. His right hand had become trapped in the blades of an industrial vacuum machine. The patient’s only complaint was pain to the third and fourth digits of his right hand. The patient had no past medical history, no prior surgeries and took only aspirin (81mg daily), folic acid, glucosamine and garlic tablets. His last tetanus shot was one year prior. The patient also denied tobacco use.

On physical examination; the patient was alert, ambulatory and talking, but in mild distress. He had a Glasgow coma scale score of 15. The only abnormal physical exam findings were as follows: Third digit of the right hand with a crush injury and avulsion of the nail bed. This was associated with complete extensor tendon deficit, with the tendon exposed on exam and a moderate amount of bleeding. The fourth digit was swollen and tender to palpation with a smaller skin defect over the dorsum of the finger with a puncture wound. There was no damage to the nail or nail bed of the fourth finger, but ecchymosis was noted to the finger pad.

**Image 1:**
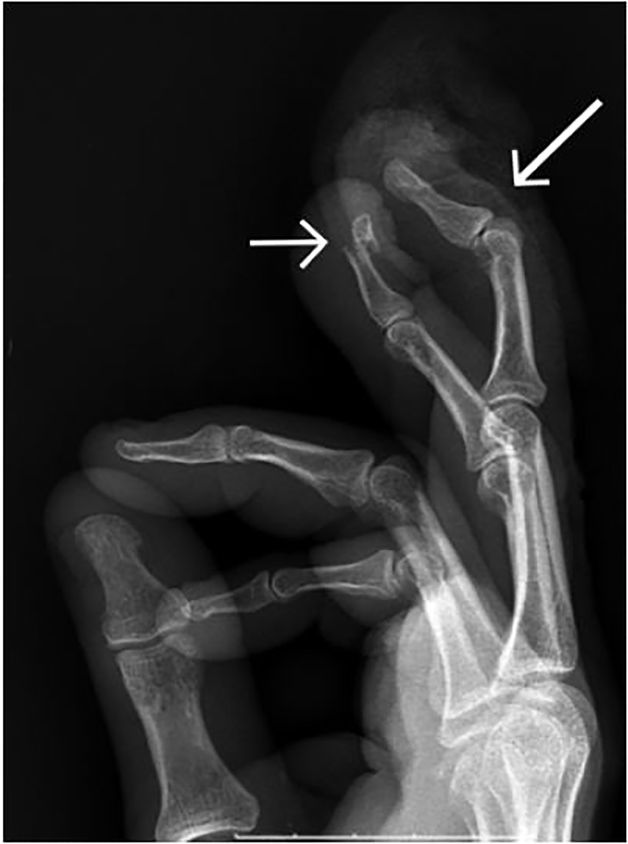
Pre-repair radiograph demonstrating a fracture (short arrow) to the fourth digit and mallet injury (long arrow) to the third digit.

A radiograph showed a mallet injury to the third finger on the right hand without associated fracture. The fourth finger demonstrated an open fracture of the distal phalynx with DIP extensor distruption (Image 1).

CPC-EM Capsule

What do we already know about this clinical entity?

Extensor tendon injury at the distal interphalangeal joint is treated with extension splinting. In cases of fracture, pinning of the digit or K-wire fixation may be required.

What makes this presentation of disease reportable?

Tissue loss overlying the extensor tendon defect prevented mechanical extension splinting. The inner trocar of an intra-osseous vascular access device was used to pin the finger in extension.

What is the major learning point?

Kirschner wire placement can be safely performed in the emergency department. The inner trocar of an EZ-IO® device was successfully used for pinning of a distal phalynx extensor tendon injury.

How might this improve emergency medicine practice?

This innovative pinning technique may allow patients to be treated in the emergency department without requiring an operating room or in a resource-limited environment.

The patient was given two grams of cefazolin intravenously. A digital block was performed with 1% lidocaine and a tourniquette applied to the base of both injured digits. Percutaneous fixation of the fourth finger was performed first to stabilize the distal fracture fragment using manual pressure and a standard 18-gauge needle. A similar procedure was then tried on the third finger to splint the digit in extension and allow for healing of the macerated tissue overlying the dorsum of the DIP, but the needle could not be advanced through the bony tissue with manual pressure. Verbal consent was obtained from the patient for use of an alternative fixation device. The needle driver for the EZ-IO^®^ system was placed inside of a sterile glove by an assistant and was used to place the inner stylet of the IOVAD needle through the tip of the finger to achieve splint fixation in extension. Percutaneous needle fixation was recommended after phone consultation with the on-call orthopedic surgeon. Both needles were placed by the emergency physician caring for the patient, using simple tactile and visual guidance.

**Image 2:**
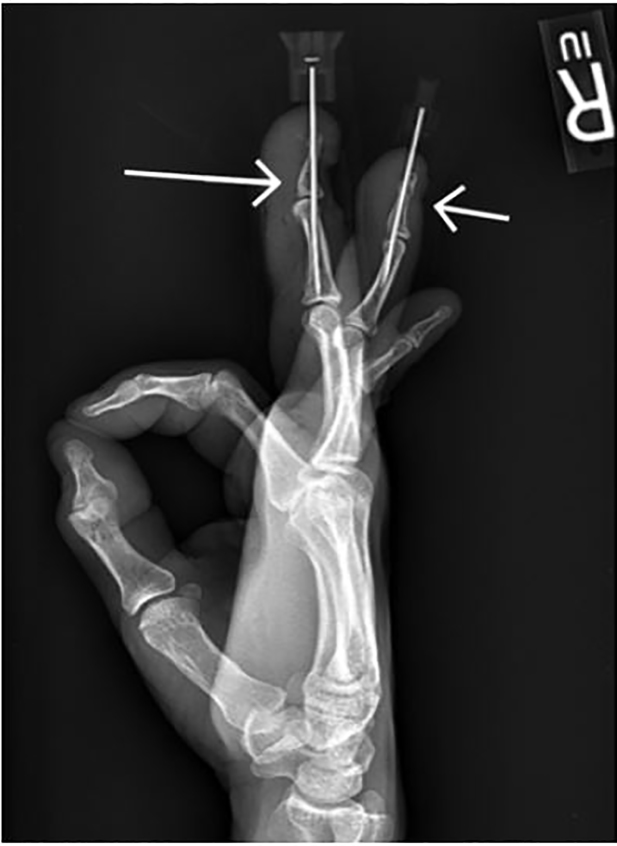
Post-repair radiograph of 3rd and 4th digits: 4th digit fracture pinned with 18-gauge needle (short arrow); 3rd digit mallet injury pinned in extension with IOVAD needle trocar (long arrow).

The patient was discharged home on cephalexin for 10 days and pain medicine with a bandage in place to protect the fingertips and exposed needles. Post-fixation films demonstrating alignment and needle comparison are shown in Image 2.

The patient followed up in the orthopedic clinic for needle removal and later physical therapy. He regained normal use of both digits.

## DISCUSSION

This is a unique case discussing the use of an IOVAD as a substitute for K-wire stabilization of mallet finger. PubMed searches using “EZ-IO, EZIO, and EZ IO” were paired individually with “arthrodesis,” “mallet,” “DIP,” “finger,” “phalanx,” and “K-wire” for a total of 18 separate search strings that yielded no results.

Non-operative treatment is successful in most mallet injuries, but mallet injury with fracture should be regarded differently. Referral to an orthopedist is required for a bony mallet finger injury, especially one that is intra-articular with greater than 30% of the articular surface involved, or if there is subluxation of the DIP. Surgical fixation does not need to occur within hours of this injury to achieve adequate results. Surgery was delayed up to eight days with excellent treatment results in some studies comparing surgical treatment strategies,^8^ and chronic injuries were repaired years later in others.^9^ Still, emergency department (ED) placement of K-wires, rather than placement in an operating room has been successfully performed without risk of osteomyelitis, even in the setting of open fractures.^10^

According to *Green’s Operative Hand Surgery,* extensor tendon injuries are classified based on involved structures and on degree and type of associated fracture.^5^ One commonly used classification method is the Doyle Classification of Mallet Injuries (Table).

Type I injuries are the most common and are amenable to non-operative treatment. Type II and type III injuries require debridement and tendon suture, if possible. If the injury is more extensive it may require skin of tendon grafting. Type IV injuries are recommended for pinning and extension splinting. The injury in this case was a type III injury.^5^

A flexion deformity usually heals well on its own with splint placement. It may need an additional surgical procedure if there is a fracture involving more than 30% of the articular surface or if the patient cannot work with the external splint. In these cases, use of K-wire fixation is a frequently implemented.^11^

The size of the K-wire used for repair is dependent upon the size of the bone and the intended use, but several authors recommend using 0.045 inch K-wire for this type of transarticular extension pinning.^2^,^8^,^12^ The15-gauge needle and solid core stylet of the IOVAD used are similar to this size. The internal diameter of a 15-gauge needle, and therefore the outer diameter of the stylet, is reported to be 0.054 inches.^13^ Also noted in the literature, although less extensively, is the use of a standard injection needle for fixation across the joint.^3^ The external diameter of the 18-gauge needle in the fourth finger seen in Image 2 is 0.050 inches. Image 2 provides a side-by-side comparison of these two techniques/devices.

In the search for an alternative to the use of K-wires it has been found that using a regular needle attached to a drill, in much the same fashion as an IOVAD, provided similar ease of insertion instead of using a K-wire.^14^ However, an electric drill is not a tool frequently found in the emergency physician’s toolkit.

The IOVAD stylet would seem to be an improvement over a standard needle because it has a solid core that seals the track, thereby reducing the risk of infection. In addition, the IOVAD comes with a drill designed to work with the included needles and may be found already in many EDs.

## LIMITATIONS

The purpose of this discussion was to provide a general overview of the primary treatment options for DIP extensor tendon injury and to provide a new way of looking at IOVADs in the ED. Because only a single patient case is available to look at this technique, generalizing the use of this procedure is left to the individual practitioner in consultation with an orthopedic surgeon. Many care providers may feel uncomfortable performing this type of procedure without orthopedic training, but the concept is presented to show other possibilities based on available resources.

## CONCLUSION

An IOVAD stylet and needle driver make a suitable and easily available alternative to formal surgical fixation with a K-wire for shallower depths, up to 45mm in length (the longest EZ-IO^®^ needle manufactured). This method was appropriate in this particular case due to the macerated tissue, which prevented the use of a supportive dorsal brace on the third digit. The fact that a standard needle could not be inserted provided the motivation to seek a new method for placement of a stabilizing piece of metal. The favorable outcome of this case was supported by excellent orthopedic follow-up and physical therapy.

## Figures and Tables

**Table:** Doyle classification of mallet injuries.

Type I: Closed injury with or without small dorsal avulsion fracture
Type II: Open injury with laceration of tendon
Type III: Open injury with loss of skin and tendon substance
Type IV: Mallet fracture
A: Transepiphyseal plate fracture (in children)
B: Hyperflexion injury with 20–50% articular surface fracture
C: Hyperextension injury with fracture of >50% articular surface and subluxation of the distal phalanx
